# Dynamics of Trust and Consumption of COVID-19 Information Implicate a Mechanism for COVID-19 Vaccine and Booster Uptake

**DOI:** 10.3390/vaccines10091435

**Published:** 2022-08-31

**Authors:** Ruben Juarez, Zheng Kang, May Okihiro, Blane K. Garcia, Krit Phankitnirundorn, Alika K. Maunakea

**Affiliations:** 1 Department of Economics and UHERO, University of Hawaii, Honolulu, HI 96822, USA; 2 Department of Pediatrics, John A. Burns School of Medicine, University of Hawaii, Honolulu, HI 96813, USA; 3 Waianae Coast Comprehensive Health Center, Waianae, HI 96792, USA; 4 Department of Anatomy, Biochemistry and Physiology, John A. Burns School of Medicine, University of Hawaii, Honolulu, HI 96813, USA

**Keywords:** COVID-19, vaccine hesitancy, information trust, information consumption

## Abstract

Vaccine hesitancy remains a significant barrier to achieving herd immunity and preventing the further spread of COVID-19. Understanding contributors to vaccine hesitancy and how they change over time may improve COVID-19 mitigation strategies and public health policies. To date, no mechanism explains how trust in and consumption of different sources of information affect vaccine uptake. A total of 1594 adults enrolled in our COVID-19 testing program completed standardized surveys on demographics, vaccination status, use, reliance, and trust in sources of COVID-19 information, from September to October 2021, during the COVID-19 Delta wave. Of those, 802 individuals (50.3%) completed a follow-up survey, from January to February 2022, during the Omicron-wave. Regression analyses were performed to understand contributors to vaccine and booster uptake over time. Individuals vaccinated within two months of eligibility (*early vaccinees*) tended to have more years of schooling, with greater trust in and consumption of official sources of COVID-19 information, compared to those who waited 3–6 months (*late vaccinees*), or those who remained unvaccinated at 6 months post-eligibility (*non-vaccinees*). Most (70.1%) early vaccinees took the booster shot, compared to only 30.5% of late vaccinees, with the latter group gaining trust and consumption of official information after four months. These data provide the foundation for a mechanism based on the level of trust in and consumption of official information sources, where those who increased their level of trust in and consumption of official information sources were more likely to receive a booster. This study shows that social factors, including education and individual-level degree of trust in (and consumption of) sources of COVID-19 information, interact and change over time to be associated with vaccine and booster uptakes. These results are critical for the development of effective public health policies and offer insights into hesitancy over the course of the COVID-19 vaccine and booster rollout.

## 1. Introduction

COVID-19 vaccination provides strong protection against severe consequences of SARS-CoV-2 infection, including hospitalizations and deaths [1,2]. The United States is one of several vaccine-producing countries where the domestic supply has far exceeded the demand since June 2021 [3]. Vaccine hesitancy (including delay or refusal) continues to undermine COVID-19 mitigation strategies [1,4] by preventing herd immunity and perpetuating viral spread. To overcome this barrier, significant efforts have been made to promote vaccine uptake. For example, multiple states have provided vaccine incentives, with mixed results of effectiveness [5,6], while vaccine mandates, focusing initially on essential workers and high-risk businesses, have increased vaccine uptake [7,8]. However, few studies have captured the nuanced changes in the attitudes and perceptions toward the COVID-19 vaccine during its rollout and the mechanisms underlying its uptake remain incompletely understood.

To accelerate population-level coverage of COVID-19 mitigation strategies involving vaccines, understanding the dynamics of vaccine and booster hesitancy is crucial. Although prior population studies demonstrated that vaccine hesitancy is associated with distrust and misinformation [9,10], none have yet reported whether these factors are directly associated with under-vaccination rates in disproportionately affected populations. Meanwhile, recent studies examining the social and behavioral characteristics of unvaccinated individuals have shown that socioeconomic factors (including education and income) impact willingness to be vaccinated, as do race, religious beliefs, and political preferences [1,11,12,13,14]. Other studies indicate that people refuse vaccination due to misinformation and inaccurate advertisements, especially on social media [15,16,17]. Together, these studies highlight the importance of trust in, and consumption of, accurate information relevant to the COVID-19 vaccine. However, how these factors interact to influence an individual’s decision about vaccination is not fully understood. This is particularly important in Hawaii, especially for the severely under-vaccinated Native Hawaiian population, where trust in the government has been historically problematic [18,19].

To gain insight into such interactions, we collected data from a survey of adults in the state of Hawaii from September to October 2021 over the course of the COVID-19 vaccine rollout. The intake survey was conducted during a significant Delta-driven surge in COVID-19 cases, and a follow-up survey from January to February 2022, during a significant Omicron-driven surge in cases. Notably, the survey-based data collected comprised common data elements as part of the National Institutes of Health (NIH) Rapid Acceleration of Diagnostics in Underserved Populations (RADx-UP) initiative [20]. The participants in our study included a large proportion of Native Hawaiian and other Pacific Islanders, who, as a whole, have been slow to initiate and complete the COVID-19 vaccine series [21]. Herein, we show for the first time the dynamics of vaccine hesitancy, as individuals become eligible for vaccination, identifying key factors that include trust in and consumption of COVID-19-relevant information, with changes in these factors by the participants over approximately four months between study enrollment and follow-up. Despite our focus on Hawaii’s population, these results confirm social factors previously implicated in vaccine hesitancy more broadly, which also translates to booster hesitancy, and offer insights into how these factors interact to influence vaccine uptake.

## 2. Materials and Methods

Taking advantage of the infrastructure developed in partnership with the NIH RADx-UP initiative at the University of Hawaii, and five federally qualified health centers within the state, we collected data from an online survey (administered via Qualtrics) of 1594 adult Hawaii residents during the COVID-19 testing rollout by the Pacific Alliance Against COVID-19 (PAAC, www.paac.info, accessed on 21 July 2022) from September to November 2021. These participants were recruited from COVID-19 testing events at community and health centers throughout the state of Hawaii, or other PAAC studies. Fifty percent of these participants (802) completed a follow-up survey from January to February 2022.

The surveys included over 100 questions related to demographics, vaccination status, and attitudes towards vaccination. For this analysis, we considered the time that individuals received the vaccine as a dependent variable, and participants were stratified into three groups based on the time (in months) they initiated the first dose of any of the three FDA-approved COVID-19 vaccines (Pfizer-BioNTech, Moderna, and Johnson & Johnson’s Janssen) after eligibility: (1) *early vaccinees*, those vaccinated within two months of eligibility; (2) *late vaccinees*, those vaccinated 3–6 months after eligibility; and (3) *non-vaccinees*, those who refused vaccination 6 or more months after eligibility, or who were only vaccinated due to a mandate by their employer or the government.

Survey questions also included how much participants trusted—and how often they used or consumed—various sources of information. The data were categorized into *official* sources of information, which included government, healthcare providers, and traditional channels of communication, such as TV, radio, and print news; and *unofficial* sources of information, which included social media channels, friends, family, acquaintances, and faith leaders (see the precise definition of these variables in Appendix A, Definition A1). Table 1 lists the demographic statistics of the data used in our analyses. We examined 17 independent variables, including education level and trust in and consumption of various information sources, as well as 4 aggregated variables, including *the official* trust index, *unofficial* trust index, *official* information consumption index, and *unofficial* information consumption index (see below and also Appendix A, Definition A1, on how these indexes were generated). All independent variables were semi-quantified, based on the descriptive rating scale, and normalized between 0 and 1. The *official/unofficial* trust and consumption indexes were computed as the average of trust and consumption within a subset of *official/unofficial* sources.

To identify factors underlying vaccine hesitancy, our regression models were separated into two parts: (1) breakdown probit regression analysis for the comparison of individuals, based on the categories of *early, late, or non-vaccinees*; and (2) longitudinal analysis of individuals.

Probit regressions were used to estimate the probability that individuals were *early, late, or non-vaccinees*. These probit regression models included two groups compared in each regression. We performed two sets of comparisons: (1) *early vaccinees* versus everyone else, and (2) *late vaccinees* versus *non-vaccinees*. The respective identification models were related as the following Equations (1) and (2):

(1) $$early_{ic}=\alpha +\beta \times trust_{ic}+X_{ic}+u_{ic};$$

and

(2) $$late_{ic}=\alpha +\beta \times trust_{ic}+X_{ic}+u_{ic},$$

where the variable $early_{ic}$ equals 1 if the participant *i* is an *early vaccinee* and 0 otherwise; the variable $late_{ic}$ equals 1 if the participant *i* is a *late vaccinee* and 0 if a *non-vaccinee*. Meanwhile, $X_{ic}$ is a control variable that includes individual-level race, sex, age, education, and the CDC social vulnerabilities of the community where the individual lives (see details at https://www.atsdr.cdc.gov/placeandhealth/svi/index.html, accessed on 21 July 2022). The trust variable index, $Trust_{ic}$, is substituted by the consumption variable, $Consume_{ic}$, when estimating the information consumption indexes. The residual term is represented by $u_{ic}$.

The longitudinal analysis focused on the changes between the entry time point and the returning time point. The regression model is as follows; Equation (3):

(3) $$Trust_{i}=\gamma \times second_{i}+\delta_{i}+u_{i},$$

where $Trust_{i}$ serves as the information trust indexes as dependent variables, which is substituted by the consumption variable, $Consume_{i}$, when estimating the information consumption indexes; $second_{i}$ is a dummy variable indicating whether the entry is from the second survey (follow-up survey) or not, and $\delta_{i}$ is the individual fixed effect. γ is the major estimator of the changes between the entries, in the two surveys, for the same participants. Moreover, the difference-in-difference analysis (as shown in Equation (4)) compares the changes of these indicators across groups (*early vaccinees* vs. *late vaccinees*) between the two surveys, thus yielding the regression model as follows; Equation (4):

(4) $$\begin{array}{c} \begin{array}{cc} Trust_{i}= & \gamma_{1}\times second_{i}\times stage_{i}+\\ \; & \gamma_{2}\times second_{i}+\gamma_{3}\times stage_{i}+\delta_{i}+u_{i}, \end{array} \end{array}$$

where $stage_{i}=1$ indicates the comparable group, and $stage_{i}=0$ indicates the control group. For instance, in the comparison of changes between early and late vaccinees, $stage_{i}=1$ if the individual is identified as a late vaccinee. Additionally, $u_{i}$ serves as the residual term in all equations above.

For our difference-in-difference analysis, the main research question focused on the comparison between early and late vaccinees between the two entries from the two surveys among participants with booster shots. However, in order to strengthen the correlation between the late vaccinees who received the booster shots and changes in the trust and/or consumption of information sources compared to early vaccinees, we performed a parallel trend analysis. A parallel trends analysis, also known as pre-trends, is the replicated work of the whole set of the analysis to the control group (i.e., *early* and *late vaccinees* without booster shots, in this case). This is always used in the identification strategies of difference-in-difference analyses to strengthen the correlation identified in the analysis to the treatment group (i.e., *early* and *late vaccinees* with booster shots, in this case), replicating the exact same analysis to those without booster shots. The stronger correlation was proven by showing the significance in the main analysis but not in the parallel trend analysis.

## 3. Results

We tested the hypothesis that education and information in official sources increase vaccine uptake and information in unofficial sources contributes to the refusal and hesitancy toward vaccines. Furthermore, the effects of these variables change across time, being associated with the decisions on booster uptakes. In particular, we sought to identify whether there was a level of trust in official or unofficial sources that associated with individuals who were vaccinated to receive the booster shot.

### 3.1. Social Factors That Contribute to the Early Adoption of COVID-19 Vaccines

**Figure 1:**
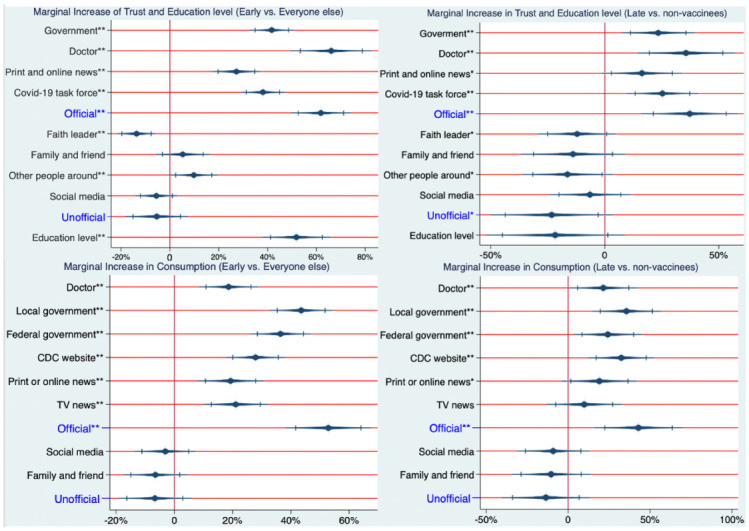
Regression results outlying the impacts of education, trust, and consumption variables on vaccination uptake. For this analysis, the impact of each variable is shown independently of the others. The values and the intervals in the graphs indicate the probability increase/decrease of being an early vaccinee or a late vaccinee, based on education, trust, and consumption variables, surrounded by 95% confidence intervals of those probabilities. Statistical significance (at * p<0.05 and ** p<0.01) is shown for each variable on trust in and consumption of various information sources.

Social factors that contribute to the early adoption of the COVID-19 vaccines (as soon as individuals become eligible) have yet to be determined. Based on prior reports examining the impacts of education, trust, and consumption of information on individual decision-making [18], we performed a probit regression analysis (Equation (1)) using data measuring these social factors collected from individual participants, comparing that of early vaccinees to all others. The results in Figure 1 (left side) suggest a significant positive impact of the level of education on early vaccinees, independent of other factors. Individuals with advanced degrees were 52% [p<0.001, 95% CI = (41%, 62%)] more likely to be early vaccinees, compared to those who lacked education beyond grades 6–12. This value is equivalent to a 5.8% increase in the probability of being an early vaccinee for each year of education past the 12th grade.

In addition to education, we found that trust in (and consumption of) information sources play a role in early vaccine uptake. Results in Figure 1 (left side) show the marginal effect (probability increase) of being an early vaccinee for each information source for an individual who increases his/her rating from the lowest rating on the 5-point descriptive rating scale (does not trust at all) to the highest rating (a great deal of trust). We note that for this analysis, the marginal effect shown in Figure 1 was performed independently of the other variables. As shown in Figure 1 (left side), individuals with a great deal of trust in doctors have a 57% [p<0.001, 95% CI = (44%, 70%)] higher likelihood of being early vaccinees relative to those who do not at all trust in doctors. Similar values are shown in Table 1 for the government [37%, p<0.001, 95% CI = (30%, 44%)], the COVID-19 task force [34%, p<0.001, 95% CI = (27%, 40%)] and print and online news [25%, p<0.001, 95% CI = (17%, 32%)].

Meanwhile, trust in unofficial information sources played less of a role than in official information sources for the early adoption of the vaccine. Trust in faith leaders had an 11% decrease [p<0.001; 95% CI = (−16%, −4.7%)] and trust in other people around had a 7.4% increase [p=0.040; 95% CI = (0.4%, 15%)] in the probability of being an early vaccinee. Trust in family and friends and social media were not associated with the probability of being an early vaccine.

Similar to trust in official information sources, consumption (in all six elements of official sources) was positively associated with the probability of being an early vaccinee. Results in Figure 1 (left side) show the marginal effect (probability increase) of being an early vaccinee for each information source for an individual who increased his/her rating from the lowest rating on the 5-point descriptive rating scale (never consumed) to the highest rating (always consumed). Individuals who always consumed information from local governments were 39% more likely [p<0.001; 95% CI = (30%, 47%)] to be early vaccinees than those who never consumed. Similar values are shown in Table 1 for other official information sources, including the federal government [32%; p<0.001; 95% CI = (24%, 40%)], the CDC website [23%; p<0.001; 95% CI = (15%, 30%)], TV news [20%; p<0.001; 95% CI = (12%, 28%)], doctors [18%; p<0.001; 95% CI = (10%, 25%)], and print or online news [14%; p=0.001; 95% CI = (5.4%, 22%)]. Consumption of unofficial information sources was not associated with early vaccination uptake.

Aggregate indexes for trust in and consumption of official and unofficial information sources were created by averaging the scores of the respective official and unofficial sources (see Appendix A Definition A1). Early vaccination was associated with the aggregate indexes of trust and consumption in official information sources. Individuals with a great deal of trust in all official sources of information were 55% [p<0.001; 95% CI = (46%, 65%)] more likely of being early vaccinees, compared to those whose levels of trust were not at all in all official sources. Similarly, individuals who always consumed all official information sources were 46% [p<0.001; 95% CI = (35%, 57%)] more likely to be early vaccinees, compared to those who never consumed any of the official information sources. Meanwhile, the aggregate indexes of trust in and consumption of unofficial information were not associated with the probability of being an early vaccinee.

### 3.2. Social Factors That Contribute to the Late Adoption of COVID-19 Vaccines

Understanding contributing factors to vaccine uptake after early vaccinees have been vaccinated allows for the development of more targeted strategies for unvaccinated individuals in the middle of a vaccine rollout. Thus, we applied a probit regression analysis (Equation (2)), to compare social factors between late vaccinees and non-vaccinees.

Similar to *early vaccinees*, we found that trust in and consumption of information sources played a role in the vaccinations of *late vaccinees*. Results in Figure 1 (right side) show the marginal effect (probability increase) of being a *late vaccinee* for each information source measured for an individual who increased his/hers rating from the lowest rating on the 5-point descriptive rating scale (do not trust at all) to the highest rating (a great deal of trust). We note that for this analysis, the marginal effect shown in Figure 1 was performed independently of the other variables. As shown in Figure 1 (right side), individuals with a great deal of trust in doctors had a 36% [p<0.001; 95% CI = (19%, 53%)] higher likelihood of being late vaccinees relative to those who did not trust at all in doctors. Similar values are shown in Figure 1 (right side) for the government [24%; p<0.001; 95% CI = (11%, 36%)], the COVID-19 task force [26%; p<0.001; 95% CI = (14%, 38%)], and print and online news [17%; p=0.013; 95% CI = (3.9%, 31%)]. We note that the results in the comparison between *late vaccinees* and *non-vaccinees* have consistent trends and similar levels of significance to the results in the comparison between *early vaccinees* and others, but with lower coefficients.

Meanwhile, trust in unofficial information sources played less of a role than trust in official information sources. Trust in faith leaders had a 13% decrease [p=0.050; 95% CI = (−26%, −0.29%)] and trust in other people around had a 18% decrease [p=0.017; 95% CI = (−34%, −3.1%)] in the probability of one being a late vaccinee instead of a non-vaccinee. Trust in family and friends and social media were not associated with the probability of being a late vaccine.

Similar to trust in official information sources, consumption in five of the elements of official sources was positively associated with the probability of being a late vaccinee. Results in Figure 1 show the marginal effect (probability increase) of being a late vaccinee for each information source for an individual who increased his/her rating from the lowest rating on the 5-point descriptive rating scale (never consume) to the highest rating (always consume). Individuals who always consumed information from local governments were 34% more likely [p<0.001; 95% CI = (18%, 50%)] to be late vaccinees than those who never consumed. Similar values are shown in Figure 1 for other official information sources, including the federal government [24%; p=0.003; 95% CI = (8.1%, 40%)], the CDC website [31%; p<0.001; 95% CI = (15%, 46%)], Doctors [21%; p=0.012; 95% CI = (5%, 37%)], and print or online news [18%; p=0.047; 95% CI = (0.2%, 36%)]. None of the elements related to the consumption of unofficial sources showed significant impacts on late vaccination.

Late vaccination is associated with the aggregate indexes of trust in and consumption of official information sources. Individuals with a great deal of trust in all official sources of information were 39% [p<0.001; 95% CI = (22%, 55%)] more likely to be late vaccinees, compared to those whose levels of trust were not at all in all official sources. Similarly, individuals who always consumed all official information sources were 41% [p<0.001; 95% CI = (20%, 62%)] more likely to be late vaccinees, compared to those who never consumed any of the official information sources. Meanwhile, the aggregate indexes of trust in and consumption of unofficial information were not associated with the probability of being an early vaccinee. Interestingly, the aggregate index of trust in unofficial information was associated with late vaccination, where individuals with a great deal of trust in all unofficial sources of information were 25% [p<0.001; 95% CI = (−46%, −5%)] less likely of being late vaccinee, compared to those whose levels of trust were ‘not at all’ in all unofficial sources. We note that the aggregate indexes of consumption of unofficial information were not associated with the probability of being a late vaccinee.

Notably, one’s level of education did not play a statistically significant role in the late adoption of vaccination, and its coefficient was negative, as shown in Figure 1 (right side).

### 3.3. Longitudinal Changes in Trust and Consumption of COVID-19 Information That Associate with the Adoption of COVID-19 Vaccine Boosters

**Figure 2:**
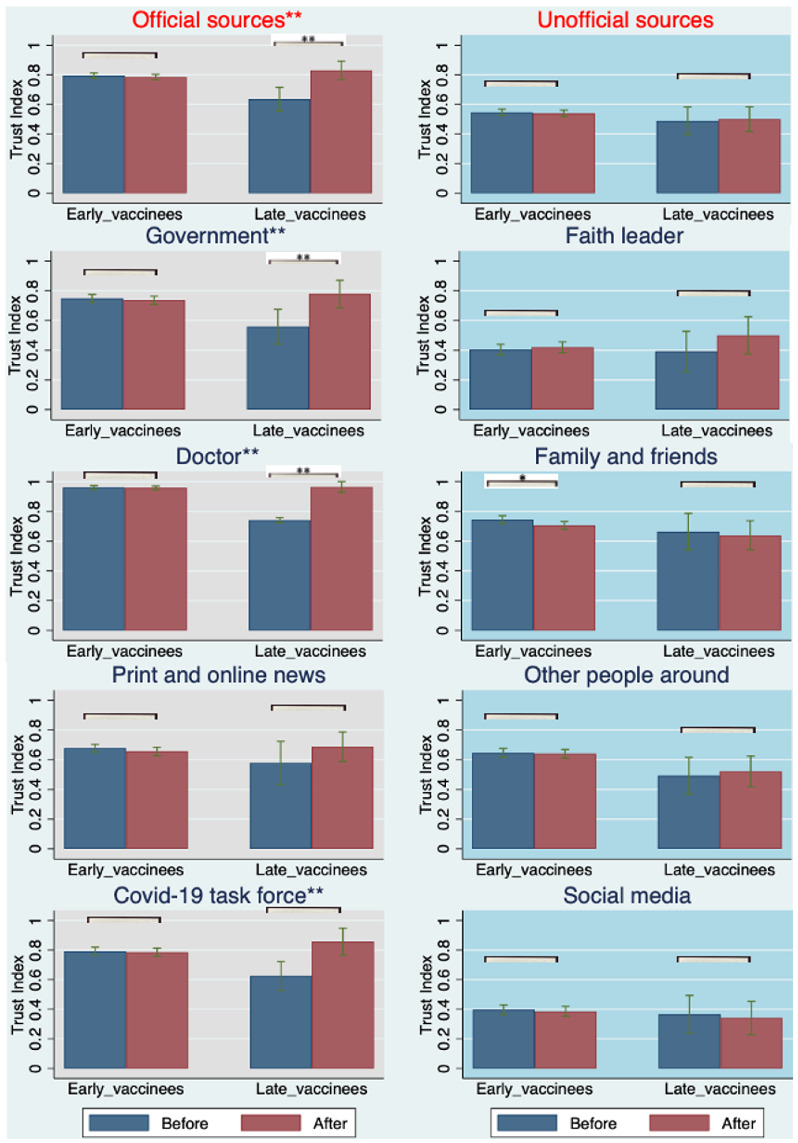
For all early and late vaccinees who received their booster shots, the graphs depict the mean values of trust indexes before and after the booster shots. For each group, separate *t*-tests were performed, and *p*-values are reported at the top of each group being compared (gray shadow: official sources; blue shadow: unofficial sources). The star levels on the titles are the comparison between early and late vaccinees on the difference before and after the booster shots. (Statistical significance at * *p* < 0.05 and ** *p* < 0.01 are shown.)

**Figure 3:**
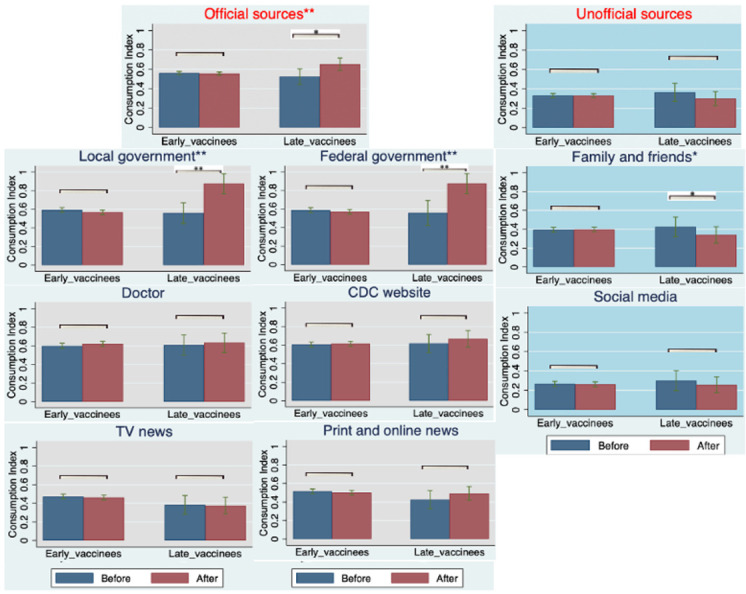
For all early and late vaccinees who received their booster shots, the graphs depict the mean values of consumption indexes before and after the booster shots. For each group, separate *t*-tests were performed, and *p*-values are reported at the top of each group being compared (gray shadow: official sources; blue shadow: unofficial sources). The star levels on the titles are the comparison between early and late vaccinees on the difference before and after the booster shots. (Statistical significance at * p<0.05 and ** p<0.01 are shown.)

How factors that contribute to the COVID-19 vaccine booster uptake relate to the trust and consumption of COVID-19 information has yet to be studied. Based on Equation (3), our longitudinal sampling provided insight into how information, trust, and consumption changed for vaccinated individuals who received boosters. Specifically, we observed changes in these metrics over a 6-month period at the individual level and associated these changes with booster uptake. Results from this regression analysis (Equation (3)) are shown in Figure 2 and Figure 3. We observed a significant increase in the level of trust in, and consumption of, official sources of information associated with the COVID-19 vaccine booster shots among late vaccinees [13%; p=0.001; 95% CI = (0.062, 0.205)] and [11%; p=0.024; 95% CI = (0.015, 0.198)], respectively. However, early vaccinees did not significantly change their levels of trust in and consumption of official sources of information [−0.007, p=0.485; 95% CI = (−0.025, 0.012)] and [−0.004, p=0.654; 95% CI = (−0.021, 0.013)]. This is likely due to the overall higher levels of trust in and consumption of official sources of information among early vaccinees at entry and prior to their booster eligibility, relative to the levels expressed by late vaccinees.

Stratifying official and unofficial information sources, more nuanced associations between trust in and consumption of various sources of information and booster uptake were observed (Figure 2 and Figure 3). First, we observed significant increases in trust in three sources of official information (government, doctors, and the COVID-19 task force) associated with COVID-19 vaccine booster shots among late vaccinees [15%; p=0.005; 95% CI = (0.052, 0.025)], [24%; p<0.001; 95% CI = (0.19, 0.29)] and [11%; p=0.077; 95% CI = (−0.013, 0.23)], respectively, while trust in print and online news did not reach significance among early vaccinees. Additionally, we observed significant changes, albeit to a smaller degree, of trust in one source of unofficial information (family and friends) associated with COVID-19 vaccine booster shots among early vaccinees [−3.3%; p=0.035; 95% CI = (−0.064, −0.002)], respectively. Furthermore, we observed significant increases in the consumption of only two of the official sources of information (local government and federal government) associated with COVID-19 vaccine booster shots among late vaccinees [24%; p=0.003; 95% CI = (0.091, 0.39)] and [26%; p=0.002; 95% CI = (0.11, 0.41)], respectively. Interestingly, the consumption of information from doctors did not show any significant change, in contrast to the comparison of trust. This may indicate that late vaccinees chose to receive booster shots because of the increased trust in their doctors, rather than increased consumption of information. Additionally, we observed a significant decrease in the consumption of only one of the sources of unofficial information (family and friends) associated with COVID-19 vaccine booster shots among late vaccinees [−14%; p=0.019; 95% CI = (−0.26, −0.025)]. This was in contrast to the comparison of trust in this source of information, which may indicate that late vaccinees received their booster shots because they consumed less information from their families and friends, rather than an increased trust in this source of information.

Finally, based on Equation (4) (difference-in-difference analysis), we observed a significantly higher degree of change in trust in and consumption of official information compared from late vaccinees to early vaccinees by [16%; p<0.001; 95% CI = (0.096, 0.217)] and [11%; p=0.004; 95% CI = (0.035, 0.178)], respectively (see Appendix D, Table A3). This result may indicate that late vaccinees were more impacted than early vaccinees by trust in and consumption of official information with respect to booster shots.

To strengthen the correlation and minimize any possible bias, we used a parallel trend analysis based on the same equation of difference-in-difference analysis (Equation (4)) for all returning participants without a booster shot under the same regression equations. All early and late vaccinees without booster shots were compared. As shown in Appendix E Table A5, there were no significant changes in the degree of trust in and consumption of official sources of information among early and late vaccinees without booster shots. Meanwhile, we considered another set of comparisons of the longitudinal changes in their trust in and consumption of official and unofficial information sources between those with booster shots and without. This set of comparisons was done both among early and late vaccinees; no significant differences were observed among early vaccinees, and significant increases were observed among late vaccinees, which was consistent with our analysis above. Collectively, these analyses support the stronger correlation between increased trust in and consumption of official information sources and booster uptake among late vaccinees.

## 4. Discussion

Vaccination has been considered one of the most effective long-term public health strategies to mitigate the severe consequences due to COVID-19 [22]. However, vaccine hesitancy remains a considerable barrier to this strategy. Therefore, identifying social factors that associate with vaccine hesitancy and how they may change over the time it takes to reach herd immunity has significant public health implications. Although our findings provide an avenue to design dynamic public health policies that include education, consumption, and trust in COVID-19 information, the content of these policies is time-dependent. Herein, we identified how social factors associated with vaccine hesitancy likely influence decisions regarding vaccine uptake over the course of a recent COVID-19 vaccine rollout.

In the first two months of COVID-19 vaccine eligibility in Hawaii, one’s level of education was a significant factor associated with vaccine uptake. However, it was not a significant factor for individuals who waited to vaccinate three or more months after being eligible. Instead, for these individuals, their decisions to vaccinate during this time were significantly associated with trust in and consumption of COVID-19 information. Indeed, trust in and consumption of official sources of COVID-19 information increased the probability of vaccination for individuals who did not vaccinate within three months of eligibility. On the other hand, trust in and consumption of unofficial sources of COVID-19 information decreased the probability of vaccinations among these individuals. Prior research has demonstrated that trust in official information sources impacted vaccination hesitancy and refusal, even before vaccine development [23], noting psychological differences between those accepting, hesitating, and refusing future vaccinations. Trust in official sources has also been shown to be an important factor for vaccine uptake [18,24].

The negative relationship between trust in unofficial sources and vaccine uptake might indicate that such sources actively discourage vaccination and potentially include factors such as the spread of misinformation (previously implicated in contributing to reduced vaccine uptake) [10,15,25]. Our results suggest that to increase the vaccinations of non-vaccinees, governments and official sources of information should complement their campaigns by appealing to unofficial information providers, including community members, faith leaders, and social media influencers.

Our findings are also relevant to COVID-19 booster hesitancy. *Early vaccinees*, already having high levels of trust in official sources, maintained their levels of trust and were boosted at high rates (70.3%). *Late vaccinees* who were boosted increased their information trust to levels similar to *early vaccinees*. In comparison, trust levels of *late vaccinees* who eschewed boosting remained similar to levels at study entry, suggesting a “threshold” of trust that must be reached for accepting follow-up boosters. Consequently, COVID-19 mitigation policies should incorporate interventions that foster trust in official sources of COVID-19 information, and promote health literacy at the early and late stages of the booster rollout.

This study has several limitations. Our survey was a convenience sample within the state of Hawaii that included all major ethnic groups and similar age distributions as the state of Hawaii. The sample collected was biased towards females and individuals with higher education levels, and adjustments were not made to match the state population. In addition, the survey may not necessarily represent the populations of other states (or the nation as a whole). Indeed, our survey collected statewide information from Hawaii, a multicultural state rich with predominantly Asian, White, Native Hawaiian, and Pacific Islander races, yet lacking significant representation from other races, including Blacks, Hispanics, and Native Americans. To minimize this limitation, we note that our robustness check (see Appendix B Table A1) showed that race was not a significant contributor in our analyses, even while this relationship is considered well established [26,27]. Another study limitation is that our model does not account for other factors, such as income, household size, the pace of vaccine development, job type/sector, risk of COVID-19 exposures, pre-existing medical conditions, and political preferences, which all may influence vaccine uptake [28,29,30]. To minimize this limitation, we controlled for other demographic variables, including race, sex, age, and education, as well as the Social Vulnerability Index (SVI) of where individuals live, which uses census data to identify and map places where a community may have more difficulty preventing human suffering and financial loss in a disaster [31]. Notably, the SVI is negatively associated with vaccine uptake in a large nationwide study [32], and we similarly found such a correlation in our data from Hawaii. However, when accounting for education level, and consumption of and trust in COVID-19 information, we found that the SVI is no longer associated with vaccine uptake.

Another limitation is our broad interpretation of official vs. unofficial sources of information. For example, while news outlets were considered official, we note that political leanings of specific news networks likely influenced COVID-19 information, while even government recommendations, at times, lacked consistency. By contrast, sources deemed unofficial could disseminate quite accurate information, depending on their own knowledge gatherings. While corrections for these confounders would likely be infeasible, the results provide insight into how and where people obtain their health information and their health promotion behaviors. As such, the data informs us about the following steps, the need to understand how people develop trust, or mistrust, in public information dissemination, and strategies to effectively promote accurate health information and COVID-19 mitigation measures.

Vaccines have the potential to decrease the adverse effects of COVID-19, with significant benefits for all. However, these benefits are only possible in a population where the vaccine is widely used. Results of this study offer insight into the nuances of vaccine hesitancy, which suggest how relevant interventions may be tailored to increase vaccine and booster uptakes.

## Figures and Tables

**Table 1:** Demographic statistics on the survey data, including descriptive statistics showing the count and percentage of participants in each vaccination stage. In addition, the numbers of returning participants in all vaccination stages are shown.

Characteristic	Early Vaccinee	Late Vaccinee	Non-Vaccinee
N=1594	$N=1150{\;}^{1}$	$N=145{\;}^{1}$	$N=299{\;}^{1}$
${}^{2}$ Sex *			
Female (*N* = 1115)	810 (73%)	103 (9%)	202 (18%)
Male (*N* = 459)	330 (72%)	42 (9%)	87 (19%)
${}^{2}$ Race **			
Caucasian (*N* = 253)	181 (72%)	17 (7%)	55 (22%)
Native Hawaiian (*N* = 598)	381 (64%)	73 (12%)	144 (24%)
Pacific Islander (*N* = 56)	33 (59%)	5 (9%)	18 (32%)
Asian (*N* = 602)	504 (84%)	40 (7%)	58 (10%)
Other (*N* = 84)	50 (60%)	10 (12%)	24 (29%)
Unknown (*N* = 1)	1 (100%)	0 (0%)	0 (0%)
${}^{2}$ Education **			
6th–12th grade (*N* = 25)	7 (28%)	7 (28%)	11 (44%)
High school (*N* = 248)	125 (51%)	43 (17%)	78 (32%)
Technical degree (*N* = 523)	355 (68%)	61 (12%)	107 (20%)
Bachelor’s degree (*N* = 435)	346 (80%)	24 (6%)	65 (15%)
Graduate degree (*N* = 337)	305 (91%)	7 (2%)	25 (7%)
${}^{2}$ Age **			
18 to 39 (*N* = 689)	418 (61%)	76 (11%)	195 (28%)
40 to 59 (*N* = 731)	574 (79%)	61 (8%)	96 (13%)
60 or older (*N* = 174)	158 (91%)	8 (5%)	8 (5%)
${}^{2}$ Returning Participants **			
Number of returning participants (percentage out of each category)	530 (46%)	100 (69%)	161 (54%)
Number of participants with booster shots (percentage out of returning participants in each category)	438 (83%)	32 (32%)	0 (0%)

^1^: n (%). Percentage out of each subgroup, unless other specified. ^2^: Pearson’s chi-squared test. Statistical significance at * *p* < 0.05, ** *p* < 0.01 are shown.

**Figure A1:**
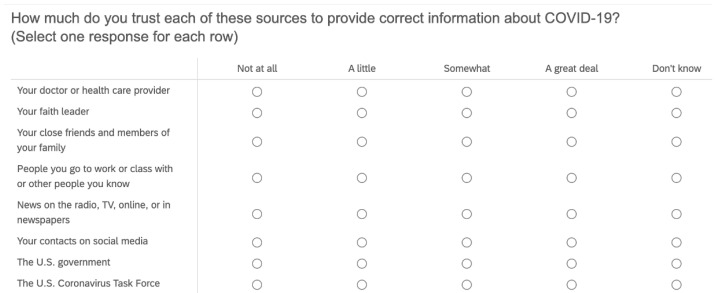
The original question in both of our surveys on trust indexes.

**Figure A2:**
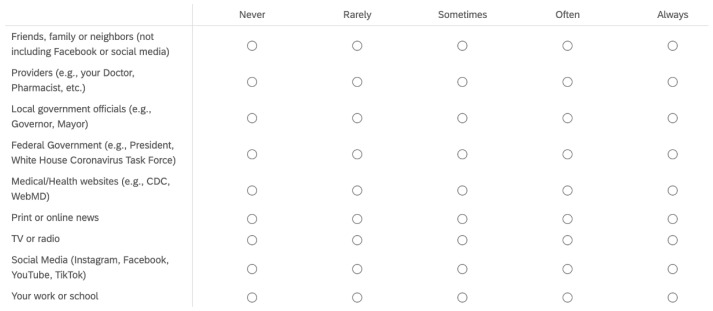
The original question in both of our surveys on consumption indexes.

## Data Availability

All data used for this project will be available (de-identified) when approved by the Waianae Coast Comprehensive Health Center Institutional Review Board upon reasonable request to the corresponding author.

## Appendix A. The Formal Definition of the Essential Variables in This Paper

Our six essential variables are vaccination stage, education level, official trust index, unofficial trust index, official information consumption index, and unofficial consumption index. These variables are generated from the survey as follows:

**Definition** **A1** (Variables generated and quantified)**.**

- *The vaccination stage reveals the decisions of the participants on getting vaccines, which is divided into three categories:*
  – ***Early Vaccinee**:**A participant is defined as an early vaccinee if and only if he/she initiated his/her first dose within 2 months of being eligible for the vaccinations;*
  – ***Late Vaccinee**:**A participant is defined as a late vaccinee if and only if he/she did not initiate his/her first dose within 2 months of being eligible for the vaccinations, but a dose was finally initiated within 6 months after being eligible;*
  – ***Non-vaccinee**:**A participant is defined as a non-vaccinee if and only if he/she never initiated a dose within 6 months after being eligible, or he/she finally initiated it but was required to do so because of the vaccine mandate declared by the State of Hawaii on 5 August 2021 and implemented on 13 September 2021.*
- ***The education level****is normalized between [0, 1] from the choices given to participants, from the lowest “6th to 8th grade” as 0, “9th to 12th grade, no diploma” as $\frac{1}{5}$, “High school graduate or GED completed” as $\frac{2}{5}$, “Some college level/Technical/Vocational degree” as $\frac{3}{5}$, “Bachelor’s degree” as $\frac{4}{5}$ to the highest “other advanced degree (Master’s, Doctoral degree)” as 1. The value is null upon the answer “I don’t want to answer this question”.*
- ***The trust index****for each source of COVID-19 information is represented by [0, 1] from a 5-point descriptive rating scale ranging from “Not at all” (0), “Not sure” ($\frac{1}{4}$), “A little” ($\frac{1}{2}$), “Somewhat” ($\frac{3}{4}$) to “A great deal” (1). Data collected from individuals allowed us to compute eight indexes: government trust, doctor trust, TV news, and other official media trust, COVID-19 task force trust, faith leader trust, family and friend trust, other people around trust and the social media (e.g., Facebook, Twitter) trust. See Figure A1 for the screenshot of the original question on trust indexes.*
- ***The official trust index****takes the average of four trust indexes for each source: government trust, doctor trust, TV news, and other official media trust, and the COVID-19 task force trust.*
- ***The unofficial trust index****takes the average of four trust indexes: faith leader trust, family and friend trust, other people around trust, and social media (e.g., Facebook, Twitter) trust.*
- ***The consumption index****for each source of COVID-19 information is represented by [0, 1] from a 5-point descriptive rating scale ranging from “Never” (0), “Rarely” ($\frac{1}{4}$), “Sometimes” ($\frac{1}{2}$), “Often” ($\frac{3}{4}$) to “Always” (1). Data collected from individuals allowed us to compute eight indexes: federal government information consumption, state government information consumption, medical providers (doctor) information consumption, TV news information consumption, healthcare website information consumption, family and friends information consumption, and social media (e.g., Facebook, Twitter) information consumption. See Figure A2 for the screenshot of the original question on consumption indexes.*
- ***The official information consumption frequency index****takes an average of six consumption frequency indexes: federal government information consumption, state government information consumption, medical providers (doctor) information consumption, TV news information consumption, healthcare website information consumption, and print or online news information consumption.*
- ***The unofficial information consumption frequency index****takes an average of two consumption frequency indexes: family and friends information consumption and social media (e.g., Facebook, Twitter) information consumption.*

## Appendix B. Statistical Result Table on the Probit Regressions on the Likelihood of Being an *Early Vaccinee* and a *Late Vaccinee*

**Table A1:** Percentage changes as marginal effects of the probit correlation between the probability of being an early/late vaccinee and the education, trust, and consumption variables, which are parallel with Figure 1. For this analysis, the impact of each variable is shown independently of the others.

Independent Variables	Early Vaccinees vs. Everyone Else—Probability ${}^{1}$	Late Vaccinees vs. Non-Vaccinees—Probability ${}^{2}$
**Official Trust**	**55% ****	**39% ****
Government trust	37% **	24% **
Doctor trust	57% **	36% **
Print and online news trust	25% **	17% *
COVID-19 task force trust	34% **	26% **
**Unofficial Trust**		**−25% ***
Faith leader trust	−11% **	−13% *
Family and friends trust		
Other people around trust	7% *	−18% *
Social media trust		
**Official Consumption**	**46% ****	**42% ****
Doctor consumption	18% **	21% *
Local government consumption	39% **	34% **
Federal government consumption	32% **	24% **
CDC website consumption	23% **	31% **
Print and online news consumption	14% **	18% *
TV news consumption	20% **	
**Unofficial Consumption**		
Family and friends consumption		
Social media consumption		
Education	52% **	
**Control Variables**		
Gender FE	Y	Y
Age	Y **	Y
Social vulnerability	Y *	Y *
Race FE	Y	Y

^1^: The marginal probability changes on each 1-point estimate elevation in the independent variables, based on the probit analysis on the comparison between early vaccinees and all others. ^2^: The marginal probability changes on each 1-point estimate elevation in the independent variables, based on the probit analysis on the comparison between late vaccinees and non-vaccinees. (Standard errors are in parentheses.) (Significance level: * *p* < 0.05, ** *p* < 0.01) (Probability shown only for significant independent variables).

## Appendix C. Statistical Results Table on the Probit Regressions on the Likelihood of Being an *Early Vaccinee* and a *Late Vaccinee*, While All Trust and Consumption Variables Are in the Same Regressions

**Table A2:** Percentage changes as marginal effects of the probit correlation between the probability of being an early/late vaccinee and the education, trust, and consumption variables, while all independent variables of individual trust and consumption variables are incorporated into the same regression. We provide this table as a robustness check to identify the variables with a strong correlation with vaccine uptake whenever other variables are controlled.

Independent Variables	Early Vaccinees vs. Everyone Else—Probability ${}^{1}$	Late Vaccinees vs. Non-Vaccinees—Probability ${}^{2}$
**Official Trust**		
Government trust	24% **	
Doctor trust	42% **	23% *
Print and online news trust		
COVID-19 task force trust		
**Unofficial Trust**		
Faith leader trust	−13% **	
Family and friends trust		
Other people around trust		−24% **
Social media trust	−15% **	
**Official Consumption**		
Doctor consumption		
Local government consumption	23% **	40% *
Federal government consumption		−33% *
CDC website consumption		23% *
Print and online news consumption		
TV news consumption		
**Unofficial Consumption**		
Family and friends consumption		
Social media consumption		
**Control Variables**		
Education	Y **	Y
Gender FE	Y	Y
Age	Y **	Y
Social vulnerability	Y *	Y *
Race FE	Y	Y

^1^: The marginal probability changes on each 1-point estimate elevation in the independent variables, based on the probit analysis on the comparison between early vaccinees and all others. ^2^: The marginal probability changes on each 1-point estimate elevation in the independent variables, based on the probit analysis on the comparison between late vaccinees and non-vaccinees. (Standard errors are in parentheses.) (Significance level: * *p* < 0.05, ** *p* < 0.01) (Probability shown only for significant independent variables).

## Appendix D. Statistical Results Table from the Longitudinal Regressions on Trust in and Consumption of Each Information Source

**Table A3:** Demographic statistics on the survey data, including descriptive statistics showing the count and percentage of participants at each vaccination stage. In addition, the numbers of returning participants at all vaccination stages are shown.

Trust in Official Information Sources	Official Overall	Government	Doctor	Print or Online News	COVID-19 Task Force
Booster—ALL ${}^{1}$	0.008 (0.009)	0.011 (0.013)	0.017 * (0.0085)	−0.012 (0.015)	0.010 (0.014)
Booster—Early vaccinees ${}^{2}$	−0.007 (0.010)	−0.003 (0.014)	−0.003 (0.008)	−0.017 (0.016)	−0.005 (0.015)
Booster—Late vaccinees ${}^{3}$	0.13 ** (0.034)	0.15 ** (0.049)	0.24 ** (0.023)	0.033 (0.088)	0.11 (0.059)
Booster difference-in-difference—Late vs. Early ${}^{4}$	0.16 ** (0.031)	0.16 ** (0.043)	0.24 ** (0.024)	0.060 (0.072)	0.14 ** (0.052)
Trust in unofficial information sources	Unofficial Overall	Faith leader	Family and friends	Other people around	Social media
Booster—ALL ${}^{1}$	−0.005 (0.011)	0.028 (0.016)	−0.032 * (0.015)	−0.002 (0.016)	−0.012 (0.018)
Booster—Early vaccinees ${}^{2}$	−0.003 (0.011)	0.029 (0.016)	0.033 * (0.016)	−0.001 (0.017)	−0.008 (0.019)
Booster—Late vaccinees ${}^{3}$	−0.011 (0.059)	0.098 (0.077)	−0.054 (0.093)	−0.011 (0.085)	−0.076 (0.085)
Booster difference-in-difference—Late vs. Early ${}^{4}$	−0.011 (0.049)	0.029 (0.067)	−0.002 (0.076)	−0.004 (0.070)	−0.062 (0.070)
Consumption of four information sources	Official Overall	Doctor	Local government	Federal government	CDC website
Booster—ALL ${}^{1}$	0.005 (0.009)	0.022 (0.016)	0.007 (0.014)	0.013 (0.014)	0.011 (0.014)
Booster—Early vaccinees ${}^{2}$	−0.004 (0.009)	0.026 (0.017)	−0.019 (0.0140	−0.013 (0.013)	0.007 (0.015)
Booster—Late vaccinees ${}^{4}$	0.11 * (0.044)	0.033 (0.078)	0.24 ** (0.072)	0.26 ** (0.074)	0.033 (0.071)
Booster difference-in-difference—Late vs. Early ${}^{4}$	0.11 ** (0.036)	−0.016 (0.063)	0.28 ** (0.058)	0.28 ** (0.061)	0.038 (0.058)
Consumption of the other four information sources	Print or online news	TV news	Unofficial Overall	Family and friends	Social media
Booster—ALL ${}^{1}$	−0.005 (0.014)	−0.017 (0.014)	−0.003 (0.010)	−0.001 (0.014)	−0.005 (0.013)
Booster—Early vaccinees ${}^{2}$	−0.012 (0.015)	−0.012 (0.015)	0.003 (0.011)	0.007 (0.014)	−0.001 (0.014)
Booster—Late vaccinees ${}^{3}$	0.065 (0.066)	0.011 (0.074)	−0.092 (0.055)	−0.14 * (0.056)	−0.043 (0.067)
Booster difference-in-difference—Late vs. Early ${}^{4}$	0.078 (0.054)	−0.018 (0.060)	−0.083 * (0.044)	−0.12 * (0.048)	−0.050 (0.053)

^1^ OLS regression on the comparison before and after the booster shot, individually for all participants with booster shots. ^2^ OLS regression on the comparison before and after the booster shot, individually for early vaccinees with booster shots. ^3^ OLS regression on the comparison before and after the booster shot, individually for late vaccinees with booster shots. ^4^ OLS regression on the comparison between early and late vaccinees regarding the comparison before and after the booster shot, individually for all participants with booster shots. (Standard errors are in parentheses) (significance level: * *p* < 0.05, ** *p* < 0.01).

## Appendix E. Results from the Difference-in-Difference Approach and the Corresponding Parallel Trends for Participants without Booster Shots

**Table A4:** Regression results outlying the longitudinal changes of returning participants with boosters on trust in and consumption of official and unofficial information sources before and after the booster shot. The four rows below focus on everyone with booster shots, every early vaccinee, every late vaccinee, and the differences between early and late vaccinees in these changes, respectively. These results are based on OLS regressions, and the coefficients are directly interpretable as unit percentage changes.

Independent Variables	Official Trust	Unofficial Trust	Official Consumption	Unofficial Consumption
Changes in trust and consumption for boosted participants between before and after the booster.				
Booster—ALL ^1^	0.008 (0.009)	−0.005 (0.011)	0.005 (0.009)	−0.003 (0.010)
Booster—Early vaccinees ^2^	−0.007 (0.010)	−0.003 (0.011)	−0.004 (0.009)	0.003 (0.011)
Booster—Late vaccinees ^3^	0.13 ** (0.034)	−0.011 (0.059)	0.11 * (0.044)	−0.092 (0.055)
Booster difference-in-difference—Late vs. Early ^4^	0.16 ** (0.031)	−0.011 (0.049)	0.11 ** (0.036)	−0.083 (0.044)

^1^ OLS regression on the comparison before and after the booster shot, individually for all participants with booster shots. ^2^ OLS regression on the comparison before and after the booster shot, individually for early vaccinees with booster shots. ^3^ OLS regression on the comparison before and after the booster shot, individually for late vaccinees with booster shots. ^4^ OLS regression on the comparison between early and late vaccinees regarding the comparison before and after the booster shot, individually for all participants with booster shots. (Standard errors are in parentheses) (Significance level: * *p* < 0.05, ** *p* < 0.01).

**Table A5:** Robustness check (parallel trends): Regression results outlying the longitudinal changes of all returning participants on trust in and consumption of official and unofficial information sources between their first and second entries. The rows focus on all returning participants, returning early vaccinees, returning late vaccinees, and the differences between early and late vaccinees, respectively. These results are based on OLS regressions controlled for individual fixed effects, and the coefficients are directly interpretable as unit percentage changes.

Independent Variables	Official Trust	Unofficial Trust	Official Consumption	Unofficial Consumption
Changes in trust and consumption for all returning participants between their first and second entries.				
Without booster—ALL	0.002 (0.014)	0.008 (0.014)	−0.019 (0.011)	0.020 (0.014)
Without booster—Early vaccinees	0.010 (0.021)	0.014 (0.023)	−0.033 (0.017)	−0.007 (0.021)
Without booster—Late vaccinees	0.027 (0.034)	0.044 (0.035)	0.013 (0.027)	0.059 (0.039)
Without booster difference-in-difference—Late vs. Early	0.011 (0.034)	0.024 (0.035)	0.048 (0.027)	0.073 * (0.037)
Returning difference-in-difference—getting booster vs. not (early vaccinees)	0.003 (0.031)	−0.040 (0.036)	0.041 (0.025)	0.014 (0.030)
Returning difference-in-difference—getting booster vs. not (late vaccinees)	0.057 (0.033)	−0.001 (0.045)	0.084 * (0.038)	−0.080 (0.041)

(Standard errors are in parentheses) (significance level: * *p* < 0.05, ** *p* < 0.01).
